# Variation in responses to photoperiods and temperatures in Japanese medaka from different latitudes

**DOI:** 10.1186/s40851-023-00215-8

**Published:** 2023-07-22

**Authors:** Ai Shinomiya, Daisuke Adachi, Tsuyoshi Shimmura, Miki Tanikawa, Naoshi Hiramatsu, Shigeho Ijiri, Kiyoshi Naruse, Mitsuru Sakaizumi, Takashi Yoshimura

**Affiliations:** 1 Division of Seasonal Biology, National Institute for Basic Biology, National Institutes of Natural Sciences, Okazaki, Aichi Japan; 2 Present Address: Laboratory of Bioresources, National Institute for Basic Biology, National Institutes of Natural Sciences, Okazaki, Aichi Japan; 3 Department of Basic Biology, School of Life Science, SOKENDAI (The Graduate University for Advanced Studies), Okazaki, Aichi, Japan; 4 Laboratory of Animal Integrative Physiology, Graduate School of Bioagricultural Sciences, Nagoya University, Nagoya, Aichi Japan; 5 Institute of Transformative Bio-Molecules (WPI-ITbM), Nagoya University, Aichi Nagoya, Japan; 6 Present Address: Department of Biological Production, Tokyo University of Agriculture and Technology, Fuchu, Tokyo, Japan; 7 Aquaculture Biology, Marine Life Science, Faculty of Fisheries Sciences, Hokkaido University, Hakodate, Hokkaido Japan; 8 Laboratory of Bioresources, National Institute for Basic Biology, National Institutes of Natural Sciences, Okazaki, Aichi Japan; 9 Department of Environmental Science, Institute of Science and Technology, Niigata University, Niigata, Japan

**Keywords:** Seasonal reproduction, Photoperiodism, Critical photoperiod, Critical temperature, Intraspecific variation, Medaka

## Abstract

Seasonal changes are more robust and dynamic at higher latitudes than at lower latitudes, and animals sense seasonal changes in the environment and alter their physiology and behavior to better adapt to harsh winter conditions. However, the genetic basis for sensing seasonal changes, including the photoperiod and temperature, remains unclear. Medaka (*Oryzias latipes* species complex), widely distributed from subtropical to cool-temperate regions throughout the Japanese archipelago, provides an excellent model to tackle this subject. In this study, we examined the critical photoperiods and critical temperatures required for seasonal gonadal development in female medaka from local populations at various latitudes. Intraspecific differences in critical photoperiods and temperatures were detected, demonstrating that these differences were genetically controlled. Most medaka populations could perceive the difference between photoperiods for at least 1 h. Populations in the Northern Japanese group required 14 h of light in a 24 h photoperiod to develop their ovaries, whereas ovaries from the Southern Japanese group developed under 13 h of light. Additionally, Miyazaki and Ginoza populations from lower latitudes were able to spawn under short-day conditions of 11 and 10 h of light, respectively. Investigation of the critical temperature demonstrated that the Higashidori population, the population from the northernmost region of medaka habitats, had a critical temperature of over 18 °C, which was the highest critical temperature among the populations examined. The Miyazaki and the Ginoza populations, in contrast, were found to have critical temperatures under 14 °C. When we conducted a transplant experiment in a high-latitudinal environment using medaka populations with different seasonal responses, the population from higher latitudes, which had a longer critical photoperiod and a higher critical temperature, showed a slower reproductive onset but quickly reached a peak of ovarian size. The current findings show that low latitudinal populations are less responsive to photoperiodic and temperature changes, implying that variations in this responsiveness can alter seasonal timing of reproduction and change fitness to natural environments with varying harshnesses of seasonal changes. Local medaka populations will contribute to elucidating the genetic basis of seasonal time perception and adaptation to environmental changes.

**Supplementary Information:**

The online version contains supplementary material available at 10.1186/s40851-023-00215-8.

## Introduction

Organisms living on Earth are subject to annual environmental changes, including changes in day length (photoperiod), temperature, and rainfall. By altering their physiology and behavior, such as reproduction, migration, hibernation, and molting, they can adapt to seasonal changes in their habitats. Solstices and equinoxes occur exactly on schedule each year, and changes in the photoperiod provide the most accurate indication of the time of year, thus enabling individuals to anticipate seasonal changes. The physiological and behavioral responses of organisms to photoperiod are called photoperiodism. The photoperiodic response of testicular growth in juncos (*Junco hyemalis*) was the first reported case of photoperiodism in vertebrates [1]. Since then, birds have been used to study photoperiodism because of their remarkable response to photoperiods. To date, it has been demonstrated that the springtime hormone thyrotropin (thyroid-stimulating hormone [TSH]) secreted from the pars tuberalis of the pituitary gland is the key regulator of seasonal reproduction in birds and mammals [2–4]. An important role played by TSH in seasonal reproduction has been also reported in fish [5]. However, how organisms sense changing photoperiods remains a mystery. It is noteworthy that the Japanese quail (*Coturnix japonica*) develops its gonads when kept under 12 h of light and 12 h of darkness (12L12D) or longer day length, whereas their gonadal development is suppressed under 11.5L12.5D conditions. Intriguingly, they can distinguish only a 30 min difference in the photoperiod [6]. The photoperiod needed to stimulate gonadal development is called the “critical photoperiod.”

Seasonal changes depend on latitude, with higher latitudes experiencing more dynamic seasonal changes, and organisms at higher latitudes facing harsher winters. Inter- and intra-species diversity in reproductive seasonality with habitat latitude has been widely observed in the animal kingdom. In vertebrates, relationships between latitude and breeding season have been investigated in many species, including mammals [7, 8], birds [9], and fish [10, 11], and a more distinct seasonality at higher latitudes has been reported. Although these reports provide important insights into latitudinal variation in reproductive seasonality, few have explicitly compared photoperiod responses under controlled environmental conditions [12]. In addition, common environmental approaches are required to assess the details of the genetic basis.

In teleost fish, in addition to photoperiod, temperature is an important cue among the environmental factors that regulate the reproductive cycle [13–15]. Recent studies have also demonstrated the effect of environmental temperature on the seasonal timing of reproduction, even in endotherms, including birds and mammals [16–18]. However, how animals perceive seasonal changes in environmental temperature and how photoperiod and temperature interact during seasonal sensing remain obscure.

The medaka, *Oryzias latipes* species complex, is a small freshwater fish native to Japan, Korea, and China. Medaka exhibits clear seasonality in reproduction [19, 20] and behavior [21, 22], and local populations in Japan range from subtropical low latitudes to cool-temperate and high latitudes across the Japanese archipelago. Experiments under artificial conditions revealed that medaka reproduction requires both long days and warm water temperatures [13]. Based on the investigation of responses of gonads under different photoperiods using populations collected in Hakodate (the northern location of Japan: cool-temperate zone) and Okinawa (the southern location of Japan: subtropical zone), a preliminary study suggested that local medaka populations from different latitudes had different responses to photoperiods [23]. Intra-specific variations in responses to changing photoperiods could contribute to investigating the genetic basis underlying photoperiod perception through forward genetic approaches using medaka [24–26].

In the present study, critical photoperiods (Experiment 1) and critical temperatures (Experiment 2), which are required for reproduction, were examined using female medaka in 11 and five populations originating from different latitudes, respectively. We also conducted a transplant experiment in high-latitude environments using medaka populations with different responses to the photoperiod and temperature. Our results showed differences in the critical photoperiod and temperature among medaka local populations. The transplant experiment in a high latitudinal environment supported the results obtained in artificial experimental environments, suggesting that variations in responsiveness to photoperiod and to temperature could alter the timing of seasonality in reproduction in natural environments. This study will contribute to elucidation of the genetic basis of responses to seasonal changes in vertebrates.

## Materials and methods

### Animals

**Fig. 1:**
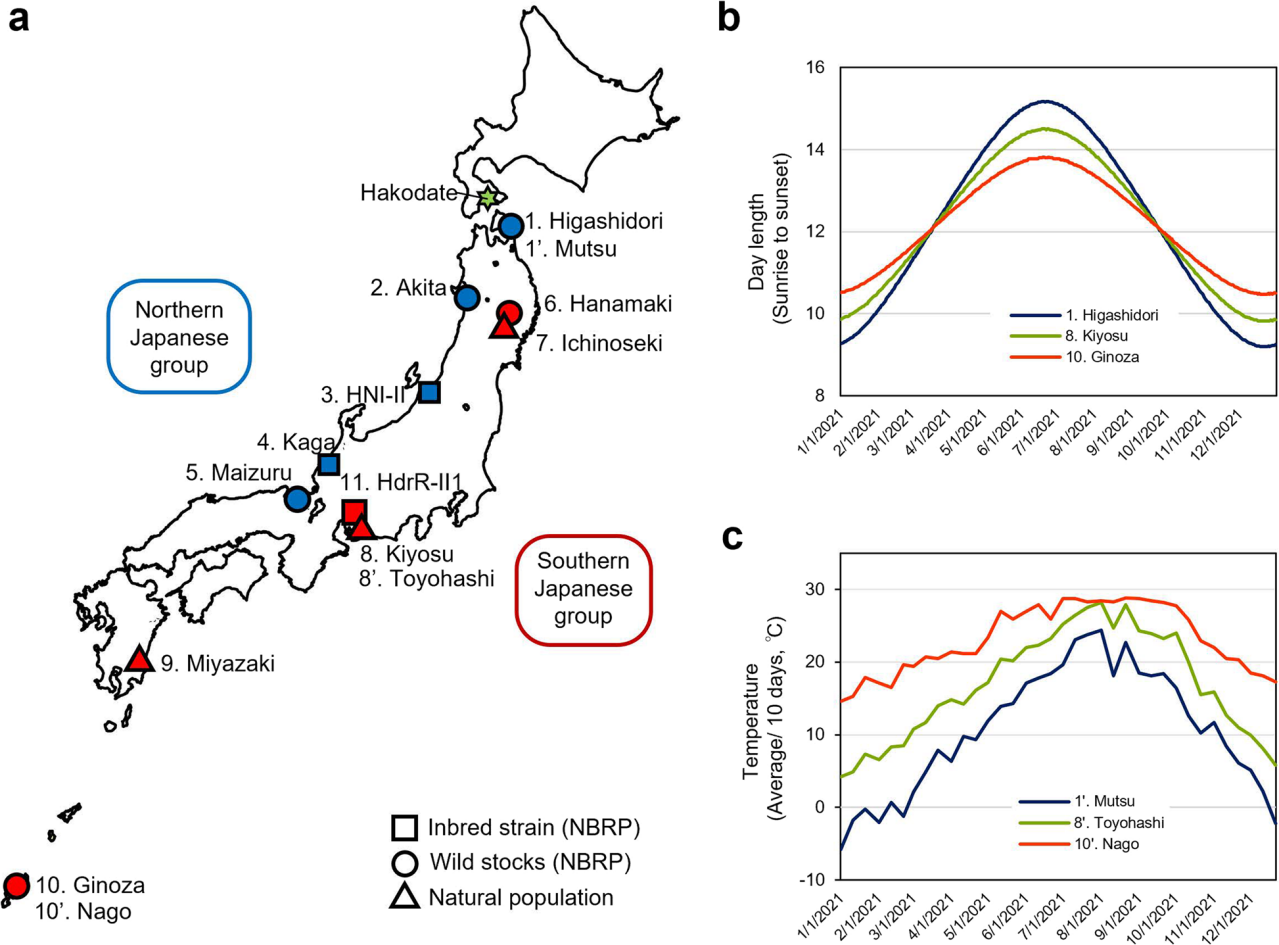
Location from which the fish used in the experiment originated. **a** Collection sites of populations used in Experiments 1–3. The numbers refer to the locations listed in Table S1. Hakodate, where Experiment 3 was conducted, is shown using a green star. **b** Differences in day length in the natural habitats. **c** Differences in temperatures in the natural habitats

Adult male and female medaka, *Oryzias latipes* species complex in five wild stocks, Higashidori (ID, WS207), Akita (ID, WS963), Maizuru (ID, WS215), Hanamaki (ID, WS223), and Ginoza (ID, WS255), and three inbred strains, HNI-II (ID, IB176), Kaga (ID, IB833), and HdrR-II1 (ID, IB178), were supplied by the NBRP Medaka (https://shigen.nig.ac.jp/medaka/). In addition, wild medaka was collected from three natural populations: Ichinoseki in Iwate Prefecture, Kiyosu in Aichi Prefecture, and Miyazaki in Miyazaki Prefecture. Medaka, which is distributed in Japan, consists of two genetically different groups: the southern Japanese group and the northern Japanese group [27–30]. The latitude and longitude location of collection sites for source individuals of each stock/strain/population (in the present study, these are referred to as “population”) and the main groups to which each population belongs are shown in Fig. 1a and Table S1.

Annual changes in day lengths and temperatures at the highest, middle, and lowest latitude locations of the populations used in this study are shown in Fig. 1b and c, respectively. Data on day length (duration from sunrise to sunset) were obtained from the National Astronomical Observatory of Japan (https://eco.mtk.nao.ac.jp/cgi-bin/koyomi/koyomiy_en.cgi), and temperature data were obtained from the Japan Meteorological Agency (https://www.data.jma.go.jp/obd/stats/etrn/index.php). Data in 2021 are shown. For temperature, data were obtained from the location closest to each collection site (1 and 1’, 8 and 8’, and 10 and 10’ in Fig. 1a and c).

### Experiment 1: Critical photoperiod investigation

Five wild stocks (Higashidori, Akita, Maizuru, Hanamaki, and Ginoza), three inbred strains (HNI-II, Kaga, and HdrR-II1), and three natural populations (Ichinoseki, Kiyosu, and Miyazaki) were used. Eggs of the G_1_ generation were obtained from the mass mating of more than three males and three females in a total of nine populations, except for the Ichinoseki population. The G_1_ offspring hatched from June to July 2013 were transferred outdoors at 2 to 4 weeks after hatching, and were maintained under natural temperature and photoperiod at the outdoor experimental field of the National Institute for Basic Biology (35.0°N, 137.2°E) in Okazaki, Aichi Prefecture. For the Ichinoseki natural population, young G_0_ fish were caught at the collection site on July 17, 2013, transferred to Okazaki, Aichi prefecture, and grown in the same natural environment as the G_1_ offspring of other populations. On days during the period January 20–February 3, 2014, five to 11 females from each population served as an initial group (IG) under outdoor conditions. IG was treated as the control group in Experiment 1. Between January 16 and February 5, 2014, in the winter season, the fish, which had been kept outdoors, were moved into a breeding room, placed at room temperature, and then transferred to experimental aquariums (MEITO system MH-R1600V, Meito Suien, Nagoya, Japan). The fish of each population were divided into five groups and treated with five different light conditions: 10 h light/14 h dark (10L), 11 h light/13 h dark (11L), 12 h light/12 h dark (12L), 13 h light/11 h dark (13L), and 14 h light/10 h dark (14L) for 3 weeks, then subjected to gonadal development analyses. The lighting hours were 7:00–17:00, 6:30–17:30, 6:00–18:00, 5:30–18:30, and 5:00–19:00, respectively (Japan standard time). The light was provided using daylight fluorescent lamps (FL15EX-D-X; NEC, Tokyo, Japan). The fish were maintained in plastic breeding tanks containing 1.5 L of water with a total of six to eight individuals, including males and females, in a water circulation aquarium system at 24 ± 0.5 °C. In HdrR-II1, because most of the females died during the experiment, the experiment was repeated using a Biomulti incubator with a Cold Cathode Fluorescent Lamp (LP-30CCFL-8CTAR; Nippon Medical & Chemical Instruments Co., Ltd., Osaka, Japan). Throughout the rearing period, fish were fed twice a day with commercial powdered foods, Hikari Labo 130 (for larvae) or Hikari Labo 450 (KYORIN, Tokyo, Japan). For fish in the outdoor experimental field, feeding was stopped after December.

### Experiment 2: Critical temperature investigation

Three wild stocks (Higashidori, Hanamaki, and Ginoza) and two natural populations (Kiyosu and Miyazaki) were used. Eggs from the G_1_ generations in Higashidori, Hanamaki, and Ginoza and eggs from the G_3_ generations in Kiyosu and Miyazaki were obtained from the mass mating of more than three males and three females. These offspring, hatched from May to June 2015, were transferred outdoors at 2–4 weeks after hatching, and were maintained under natural temperature and photoperiod in the outdoor experimental field of the National Institute for Basic Biology in Okazaki, Aichi prefecture. On January 8, 2016, six–nine females from each population served as the initial group (IG) under outdoor conditions. IG was treated as the control group in Experiment 2. Between January 6 and 8, 2016, during the winter season, the fish were moved into a breeding room, divided into four groups, and treated at four different temperatures: 14 ± 0.5 °C, 16 ± 0.5 °C, 18 ± 0.5 °C, and 20 ± 0.5 °C, in experimental aquariums (MEITO system MH-R1600V, Meito Suien, Nagoya, Japan) for 16, 18, and 20 °C, or in custom-made aquariums (Iwaki, Osaka, Japan) for 14 °C. They were maintained in a water circulation aquarium system under long photoperiodic conditions (14L10D). The light was provided using daylight fluorescent (MEITO system) or LED (Iwaki-custom-made aquarium) lamps. These fish were maintained under the same conditions for 4 weeks and then subjected to gonadal development analyses. In the Higashidori, Hanamaki, and Kiyosu populations, gonadal development at 6 weeks was also analyzed. Throughout the rearing period, fish were fed twice a day with commercial powdered foods, Hikari Labo 130 (for larvae) or Hikari Labo 450 (KYORIN, Tokyo, Japan). For fish in the outdoor experimental field, feeding was stopped after December.

### Experiment 3: Gonadal development in a natural cool-temperate and high latitude environmental condition

To mimic the northernmost natural environment of the medaka habitat, where winter environments are harsh and spring is the latest to arrive, and to compare the timing of gonadal development in spring between populations with different critical photoperiods and critical temperatures, two medaka wild stocks were set up in an outdoor experimental field at Hokkaido University in Hakodate, Hokkaido Prefecture (Fig. 1a). Higashidori, which originates from the northern limit region, and Hanamaki, which has different critical photoperiods and critical temperatures from Higashidori in the experimental environment (Experiments 1 and 2), were used in Experiment 3. Sibling eggs were obtained from Higashidori and Hanamaki, and larvae hatched from June to September 2020 were reared in experimental aquariums (custom-made aquariums; Iwaki, Osaka, Japan) for 7 to 10 months. The fish were transferred and maintained under natural temperature and photoperiod conditions in the outdoor experimental field of the National Institute for Basic Biology in Okazaki, Aichi Prefecture, from May to December 2021. On December 21–23, 2021, the fish were transferred to the outdoor experimental field of Hokkaido University (41.8°N, 140.7°E) in Hakodate and exposed to the natural photoperiod. The 54 L rearing tanks for these fish were placed inside a large 1-ton container. The water temperatures of the rearing tanks were recorded using temperature loggers (TR-52i; T&D Corporation, Matsumoto, Japan). To equalize the water temperature between the rearing tanks, the outer tank was filled with water that was agitated by a blower. To prevent severe freezing of the rearing tanks, groundwater was allowed to flow into the outer tanks, and ice frozen on the surface of the rearing tanks was checked and broken off every morning from December 24, 2021, to February 24, 2022. The average daily water temperature in these rearing tanks during that period ranged from 3.9 °C to 7.6 °C, and the difference between tanks was maintained at an average of less than 1 °C. By stopping the inflow of groundwater into the external tanks on February 25, 2022, the tank water was exposed to natural temperature conditions. On April 5, 2022, six females from Higashidori and Hanamaki served as an initial group (IG) under outdoor conditions. The IG was used as the control. From April 19 to June 21, 2022, four to eight females from each population were subjected to gonadal development analysis at 1- or 2-week intervals. Fish were fed once a day with Hikari Labo 450 (KYORIN, Tokyo, Japan) from April 20, 2022.

### Measurement of gonadal development

The fish were anesthetized with 0.05% 3-aminobenzoic acid ethyl ester methanesulfonate (MS222), water was removed from the body surface by blotting with paper towels, and their body weight (BW) was measured using an electronic balance (PRACTUM213-1S, Sartorius, Germany) with an accuracy of 1 mg. After cutting off the head with a scalpel, the abdomen was opened with microscissors, and the gonads were dissected with sharp tweezers under a stereo microscope and weighed on an electronic balance (Balance XS105, Mettler Toledo, Switzerland) with an accuracy of 0.01 mg. The gonadosomatic index (GSI) was obtained according to the following equation:

$$GSI\hspace{0.17em}=\hspace{0.17em}(Gonadal\ weight / BW) \times 100.$$

For Experiment 3, electronic balances (Balance XSR105DUV, Mettler Toledo, Switzerland) were used to measure the gonadal weight.

### Statistical analysis

For multiple comparisons among all experimental groups in Experiment 1 and Experiment 2, one-way ANOVA and Tukey–Kramer tests were conducted using the statistical software BellCurve for Excel version 4.02 (Social Survey Research Information Co., Ltd., Japan). In Experiment 3, one-way ANOVA and Dunnett’s post hoc tests were performed to estimate the gonadal development start date. A two-way ANOVA and Tukey tests were performed to examine the effect of dates and populations on gonadal development and the interaction of these two factors using BellCurve for Excel.

## Results

### Differences in critical photoperiods among medaka populations

**Fig. 2:**
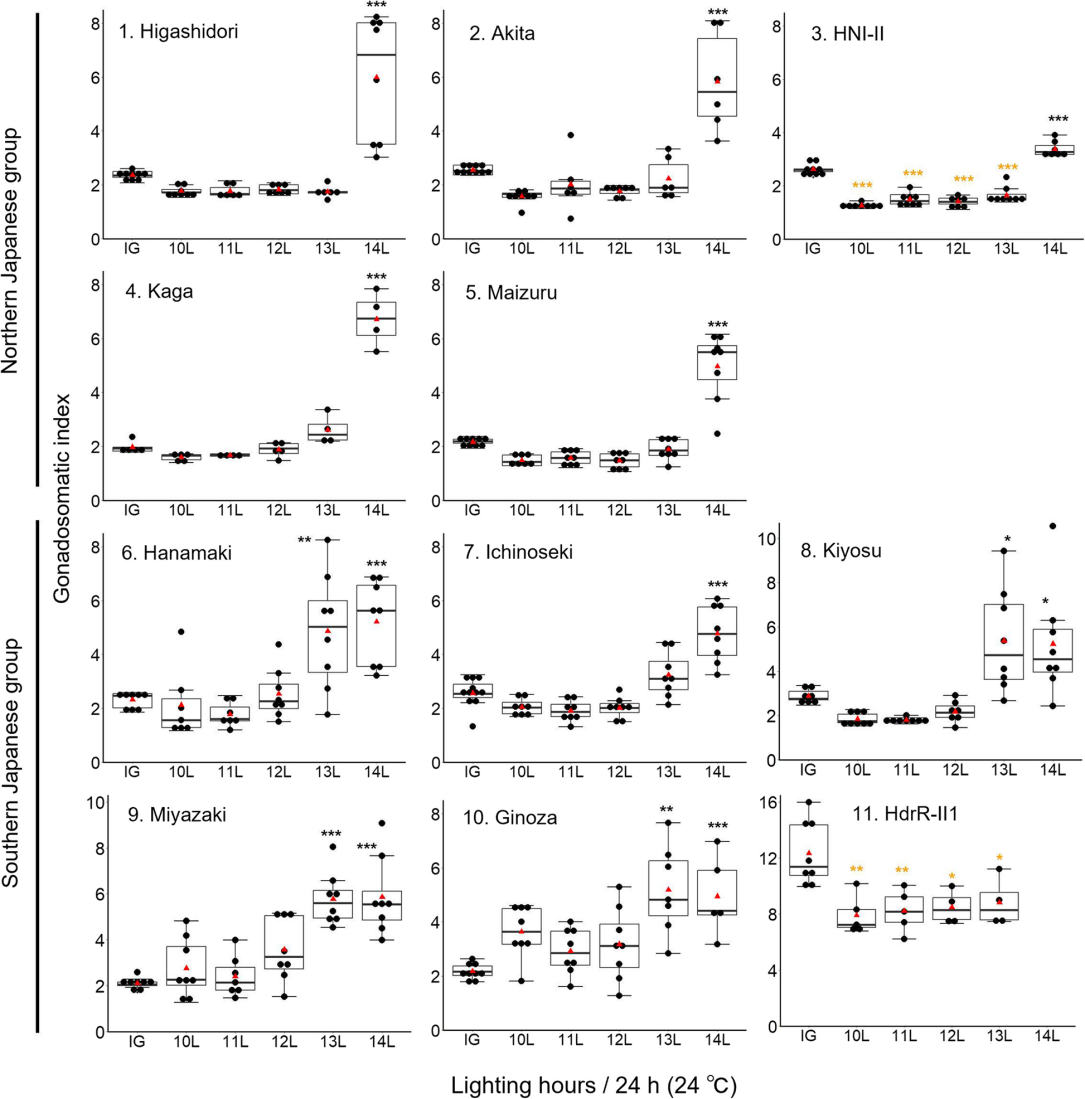
Box plot with dot plot for the GSI values of females under five different photoperiod conditions and the winter conditions (initial group, IG) in 11 populations. The raw GSI values are shown in Table S2. A red triangle indicates the mean value in each group. The fish were kept at 24 ± 0.5 °C for 3 weeks. Black and orange asterisks indicate significantly higher and lower values compared to IG, respectively. *p < 0.05, **p < 0.01, ***p < 0.001. The raw statistical results of one-way ANOVA and Tukey–Kramer tests are shown in Tables 1 and S3

The gonadosomatic index (GSI) is an essential parameter for investigating fish reproduction and serves as an indicator of the reproductive seasonality of fish species including medaka [19, 20, 23]. In the data analyses of Experiment 1, to avoid underestimating GSI by including immature individuals that did not reach a proper size at maturity, individuals weighing 200 mg or more were used. The GSI values of the females under different photoperiods in the 11 populations are shown in Fig. 2 and Table S2. The statistical results of one-way ANOVA and Tukey–Kramer tests are shown in Tables 1 and S3, respectively. The GSI under the 14L condition in all five populations from the Northern Japanese group was significantly higher than that in IG (Fig. 2, Table S3). In addition, apparent differences were observed in the individual data between the 10–13L and 14L conditions (Fig. 2, Table S2). In HNI-II, although the GSI in the 14L condition was significantly higher than that in IG, the GSI values in the four conditions of 10L to 13L were significantly lower than that in the IG (*p* < 0.001, Fig. 2, Table S3).

**Table 1:** F values and* p*-values of one-way ANOVA on GSI in Experiment 1

	Higashidori	Akita	HNI-II	Kaga	Maizuru	Hanamaki	Ichinoseki	Kiyosu	Miyazaki	Ginoza	HdrR-II1
F value	21.56	22.21	95.35	74.84	44.57	10.25	24.13	10.47	15.40	7.83	7.16
*p*-value	< 0.001^***^	< 0.001^***^	< 0.001^***^	< 0.001^***^	< 0.001^***^	< 0.001^***^	< 0.001^***^	< 0.001^***^	< 0.001^***^	< 0.001^***^	< 0.001^***^

^***^p < 0.001

In contrast, the GSI of the 13L and 14L conditions in four of the six populations from the Southern Japanese group, Hanamaki, Kiyosu, Miyazaki, and Ginoza, were significantly higher than those in the IG (Fig. 2). In the Ichinoseki natural population, although a significant difference between GSI in the 13L and IG groups was not observed, the GSI of more than half of the individuals in the 13L condition was higher than that of individuals in the 10L, 11L, and 12L conditions (Fig. 2). The GSI in the 13L group was significantly higher than that in the 10L, 11L, and 12L groups (Table S3, *p* < 0.01). The individual GSI data of IG in the Miyazaki and the Ginoza populations were from 1.72 to 2.58 and from 1.79 to 2.63, respectively (Fig. 2, Table S2). In contrast, the GSI of certain individuals in the 10L, 11L, and 12L groups in both populations was higher than that in the IG, which was close to or greater than 4.0 (Table S2), and fertilized eggs were obtained from the 11L group in Miyazaki and the 10L group in Ginoza. These results show that fish in Miyazaki and Ginoza can reproduce even under short-day conditions.

The results for HdrR-II1 females were quite different from those of other populations. The average GSI in the IG group of HdrR-II1 showed the highest value (9.98–16.00) (Fig. 2, Table S2). Observation using a stereo microscope revealed numerous large oocytes in the ovaries. Although these GSI values in the 10–13L groups were significantly lower than those in the IG, all of the GSI values in all of these groups were high (6.22 to 11.22, Table S2). Fertilized eggs were obtained under all of the photoperiodic conditions, indicating that HdrR-II1 can reproduce under short-day conditions.

### Differences in critical temperatures among medaka populations

**Fig. 3:**
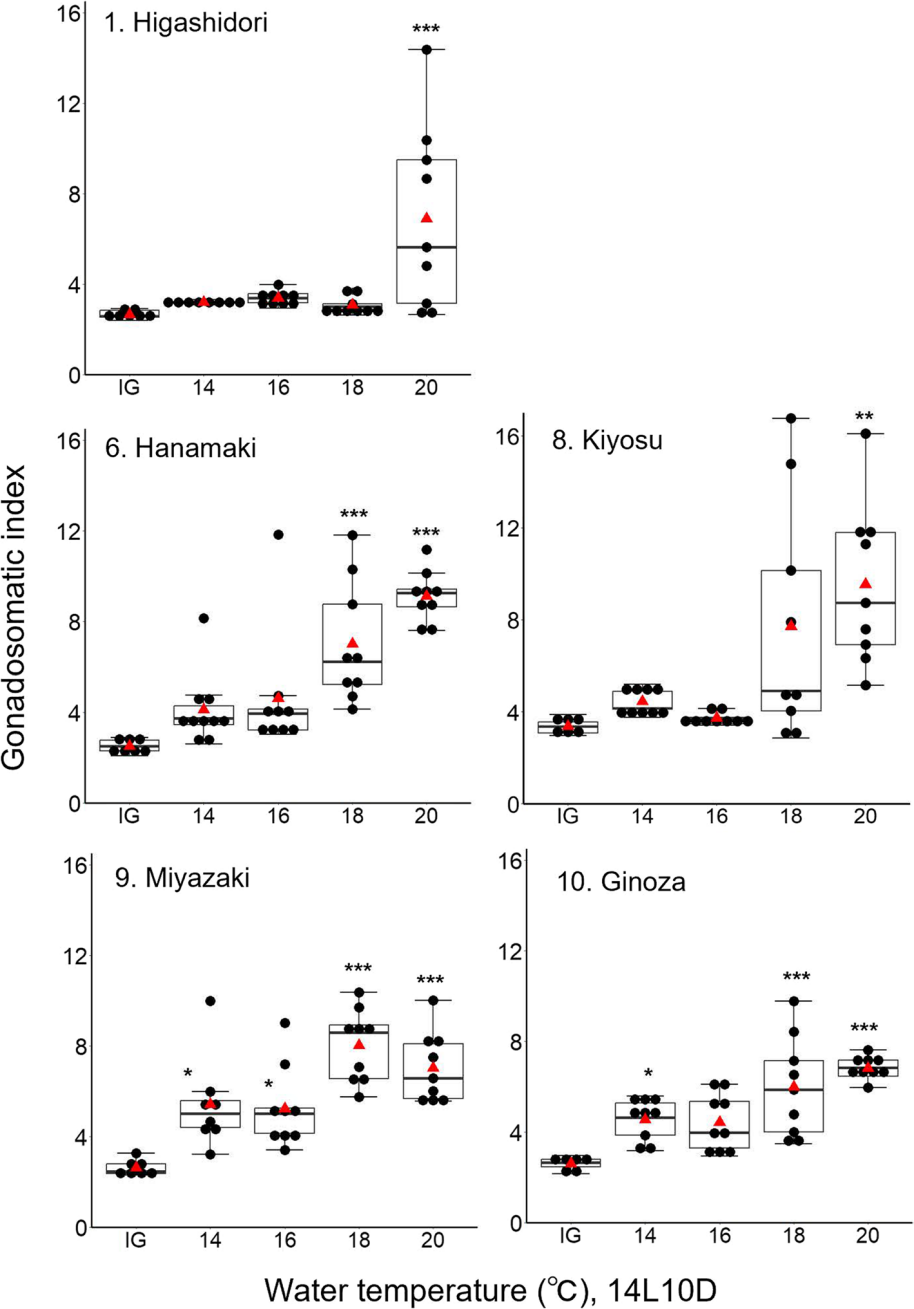
Box plot with dot plot for the GSI values of females under long photoperiodic conditions (14L10D) at four different temperature conditions for 4 weeks and under the winter conditions (initial group, IG) in five wild-derived populations. The raw GSI values are shown in Table S4. A red triangle indicates the mean value in each group. Black asterisks indicate significantly higher values compared to IG. *p < 0.05, **p < 0.01, ***p < 0.001. The raw statistical results of one-way ANOVA and Tukey–Kramer tests are shown in Tables 2 and 3

The average GSI values of females under different temperatures for 4 weeks in the five populations are shown in Fig. 3 and Table S4. In Higashidori, the average GSI in the 20 °C group was significantly higher than that in IG (Fig. 3, Tables 2 and 3). In Hanamaki, the average GSI in the 18 °C and 20 °C groups was significantly higher than that in IG (Fig. 3, Tables 2 and 3). In Kiyosu, the average GSI in the 20 °C group was significantly higher than that in IG; the GSI in the 18 °C group was not significantly higher than in IG, but significantly higher than that in the 16 °C group, and four of the nine individuals in the 18 °C group had clealy higher GSI values than the IG, 14 °C, and 16 °C groups (Fig. 3, Table 3). The GSI in all four temperature groups (14, 16, 18, and 20 °C) in Miyazaki and the GSI in the three groups (14, 18, and 20 °C) in Ginoza were significantly higher than that in IG (Fig. 3, Tables 2 and 3). Because the rate of gonadal development is generally slow at lower temperatures in the normal temperature range, a 4-week analysis would be considered insufficient in certain cases to determine gonadal development at lower temperatures. To determine whether the GSI differences detected among the populations depended on differences in sensitivity to temperature or critical temperatures of reproduction, the gonads of the Higashidori, Hanamaki, and Kiyosu populations were analyzed after 6 weeks. At 6 weeks, the temperature differences were clearer than those at 4 weeks in all populations analyzed, whereas the tendency for the responses to each temperature did not change (Figs. 3 and S1 and Tables S4 and S5). Although a significant difference between the GSI average in the 18 °C and IG groups was not observed in Higashidori, the GSI in the 18 °C group was significantly higher than that in the IG group for both Hanamaki and Kiyosu (Fig. S1 and Tables S6 and S7). The GSI in the 16 °C group of the Hanamaki population was significantly higher than that in the IG group at 6 weeks.

**Table 2:** F values and *p*-values of one-way ANOVA on GSI at 4 weeks in Experiment 2

	Higashidori	Hanamaki	Kiyosu	Miyazaki	Ginoza
F value	7.10	14.69	7.32	12.91	11.88
*p*-value	< 0.001***	< 0.001***	< 0.001***	< 0.001***	< 0.001***

^***^p < 0.001

**Table 3:** *p*-values of Tukey–Kramer tests on GSI at 4 weeks in Experiment 2

Level1	Level2	Higashidori	Hanamaki	Kiyosu	Miyazaki	Ginoza
IG	14 °C	0.9834	0.4708	0.9531	0.0143^*^	0.0479^*^
IG	16 °C	0.9431	0.2310	0.9993	0.0196^*^	0.0753
IG	18 °C	0.9924	< 0.001^***^	0.0547	< 0.001^***^	< 0.001^***^
IG	20 °C	< 0.001^***^	< 0.001^***^	0.0024^**^	< 0.001^***^	< 0.001^***^
14 °C	16 °C	0.9995	0.9813	0.9839	0.9994	0.9994
14 °C	18 °C	1.0000	0.0210^*^	0.1468	0.0156^*^	0.1479
14 °C	20 °C	0.0027^**^	< 0.001^***^	0.0058^**^	0.2520	0.0049^**^
16 °C	18 °C	0.9971	0.0913	0.0466^*^	0.0064^**^	0.0939
16 °C	20 °C	0.0035^**^	< 0.001^***^	0.0013^**^	0.1468	0.0026^**^
18 °C	20 °C	0.0013^**^	0.1770	0.6708	0.6926	0.6276

^*^p < 0.05

^**^p < 0.01

^***^p < 0.001

### Ovarian development in Higashidori and Hanamaki under natural environmental conditions

**Fig. 4:**
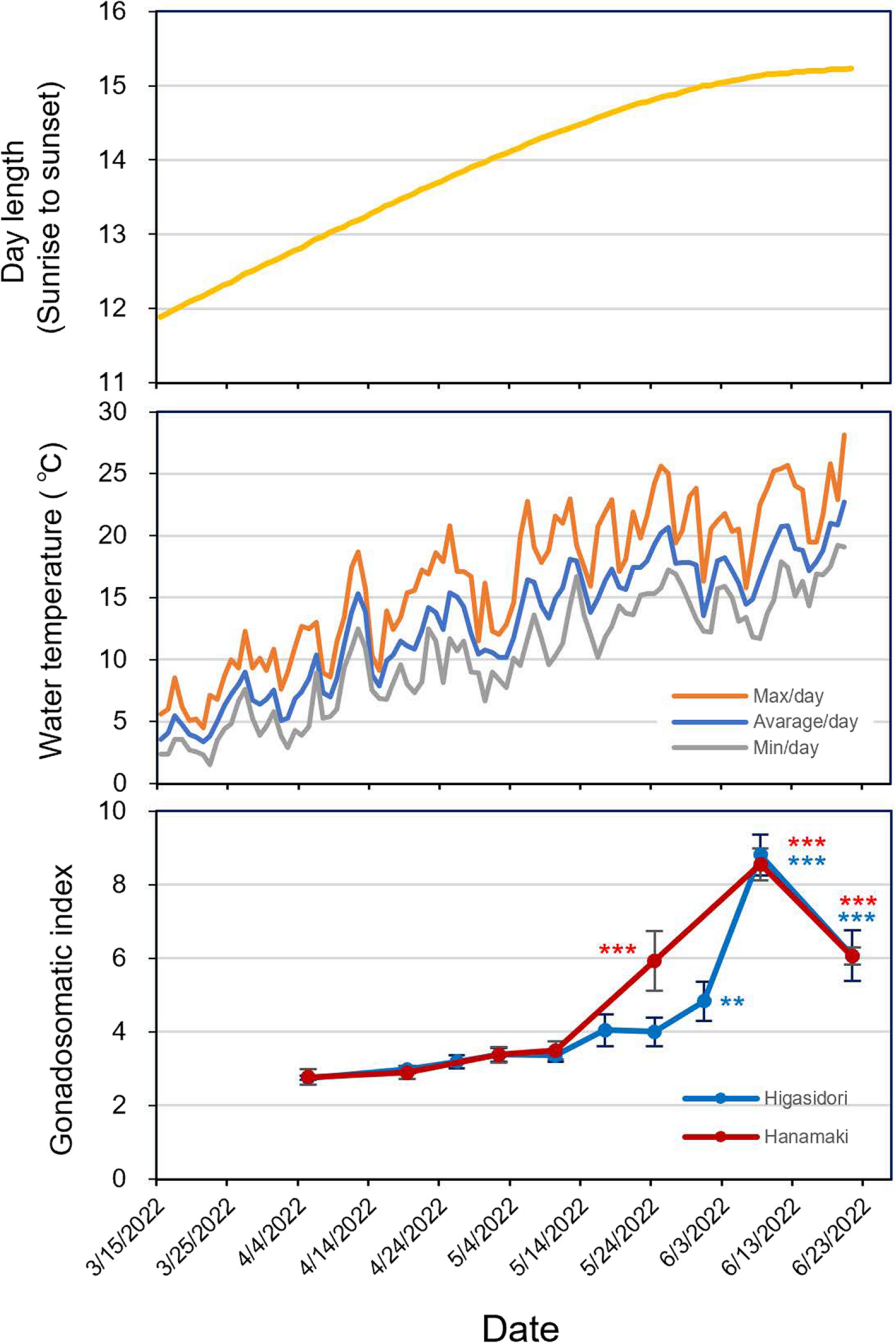
Day length (top), water temperature (middle), and gonadal development of the Higashidori and Hanamaki populations (bottom) at the Hakodate outdoor experimental field. The raw data of the day length and the temperature and the GSI values are shown in Tables S8 and S9. (Bottom) Mean ± SEM, *n* = 4–8. **p < 0.01, ***p < 0.001 by Dunnett’s test (vs. Gonadosomatic index on April 5). The raw statistical results of one-way ANOVA and Dunnett’s post hoc tests are shown in Tables 4 and 5

From December 21–23, 2021, 96 females and 54 males in Higashidori and 74 females and 32 males in Hanamaki were transferred from the Okazaki field to the Hakodate field, and then maintained there from winter to spring (see Materials and Methods for details). On April 5, 2022, the surviving fish were counted: 86 females and 49 males were observed to be alive in Higashidori, and 50 females and 22 males were observed to be alive in Hanamaki. Survival rates were 90% in Higashidori and 68% in Hanamaki. The changes in day length, water temperature, and average GSI of female medaka in Higashidori and Hanamaki are shown in Fig. 4 and Tables S8 and S9. In Hanamaki, the average GSI on May 24 was significantly higher than that on April 5 (*p* < 0.001, Fig. 4, and Tables 4 and 5). However, in Higashidori, the first significant difference in GSI compared to April 5 was observed on May 31, a week later than that in Hanamaki (*p* = 0.010, Fig. 4, and Tables 4 and 5). On June 8 and 21, all individuals in both populations had high GSI values (Fig. 4 and Table S8). To examine whether there is an interaction between the date (April 5 [IG], May 24 [the date gonadal size increase was detected in Hanamaki]), and June 8 (the date gonadal size increase was detected in both Higashidori and Hanamaki) and populations (Higashidori and Hanamaki) on GSI, a two-way ANOVA was conducted. The results showed a significant difference in the date factor (*p* < 0.001) and the interaction effects (*p* = 0.040) (Table 6). Tukey tests demonstrated the significant differences between Higashidori and Hanamaki on May 24 (*p* = 0.040, Table 7).

**Table 4:** F values and *p*-values of one-way ANOVA on GSI in Experiment 3

	Higashidori	Hanamaki
F value	22.76	30.84
*p*-value	< 0.001^***^	< 0.001^***^

^***^p < 0.001

**Table 5:** *p*-values of Dunnett’s post hoc tests on GSI in Experiment 3

Level1	Level2	Higashidori	Hanamaki
4/5	4/19	0.777	0.810
4/5	4/26	0.613	-
4/5	5/2	0.439	0.412
4/5	5/10	0.477	0.355
4/5	5/17	0.062	-
4/5	5/24	0.075	< 0.001^***^
4/5	5/31	0.001^**^	-
4/5	6/8	< 0.001^***^	< 0.001^***^
4/5	6/21	< 0.001^***^	< 0.001^***^

^**^p < 0.01

^***^p < 0.001

**Table 6:** F values and *p*-values of two-way ANOVA on GSI in on Apr. 5, May 24, and June 8 in Experiment 3

Factor	F value	*p*-value
Population: 1	2.50	0.124
Date: 2	94.36	< 0.001^***^
1^*^2	3.56	0.040^*^

^*^p < 0.05

^***^p < 0.001

**Table 7:** *p*-values of the Tukey test on GSI on Apr. 5, May 24, and June 8 in Experiment 3

Factor	Level1	Level2	*p*-value
April 5	Higashidori	Hanamaki	0.969
May 24	Higashidori	Hanamaki	0.005^**^
June 8	Higashidori	Hanamaki	0.691
Higashidori	April 5	May 24	0.083
Higashidori	April 5	June 8	< 0.001^***^
Higashidori	May 24	June 8	< 0.001^***^
Hanamaki	April 5	May 24	< 0.001^***^
Hanamaki	April 5	June 8	< 0.001^***^
Hanamaki	May 24	June 8	< 0.001^***^

**p < 0.01

^***^p < 0.001

## Discussion

The present study showed that there are differences in critical photoperiods and critical temperatures for the beginning of seasonal reproduction among medaka local populations. There were differences between the Northern and the Southern Japanese groups and between populations derived from low latitudinal regions and other regions within the Southern Japanese group. Since the individuals used in this study had been reared under the same environmental conditions, it is clear that the differences in critical photoperiods and critical temperatures among local medaka populations are due to genetic differences. The wild stocks provided by NBRP Medaka were maintained for more than 10 years in an outdoor breeding environment at Niigata University (38°N, 139°E) for research purposes. The present findings that the five wild stocks (Table S1) provided by NBRP showed differences in photoperiod and temperature responses suggest that the traits controlling seasonal reproduction did not change to adapt to the new environment during a few decades. Wild medaka is distributed over a wide range of latitudes from subtropical Okinawa Prefecture (26°N) to cool-temperate Aomori Prefecture (41°N) in Japan, and their genetic population structure has been elucidated from studies on the nucleotide sequences of the complete cytochrome *b* gene and genome-wide diversity by genotyping-by-sequencing [29, 30]. More than 80 wild stocks covering almost all medaka habitats in Japan are available from NBRP Medaka. It is worth noting that these wild medaka stocks are excellent materials for studying environmental adaptations.

### Critical photoperiod

In Experiment 1, all five populations belonging to the Northern Japanese group had critical photoperiods between 13 and 14L, which was longer than that in all wild-derived populations belonging to the Southern Japanese group with critical photoperiods under 13L (Figs. 1a and 2). Although the collection sites of Hanamaki and Ichinoseki were located at higher latitudes than the sites of HNI-II, Kaga, and Maizuru in the Northern Japanese group, they showed shorter critical photoperiods than those in the Northern Japanese group (Fig. 2). The differences in the critical photoperiods between the Northern and Southern Japanese groups were more consistent with phylogenetic differences [29, 30] than with differences in the latitude of habitats of the populations (Fig. 1a). Because the critical photoperiod was examined at a resolution of 1 h, the present results demonstrated that medaka can perceive the differences between photoperiods of at least 1 h. Although we could not detect differences in the critical photoperiod among populations belonging to the Northern Japanese group, a more precise examination might distinguish differences among their critical photoperiods.

In HNI-II, the average GSI was significantly lower than that in IG under photoperiod conditions shorter than 14L (Fig. 2, orange asterisks). These results suggest that long days promote reproduction, while short days strongly suppress reproduction and decrease ovarian size at an appropriate temperature. Fujimoto et al. [20] reported seasonal changes in GSI in a natural Aomori population (Kuwabara, Aomori City, 40.8°N, 140.7°E). The GSI values decreased sharply in August and then increased slightly over the next few months. As the water temperature in August was similar to that in July, the suppression of reproduction appeared to be caused by a decrease in day length. The suppression of reproduction and decrease of gonad size induced by decreasing day length would be a process that would proceed quickly in the natural environment during late summer. This may be an effective way to prepare for a cold season without wasting energy.

In the Southern Japanese group, Hanamaki and Kiyosu had a critical photoperiod between 12 and 13L (Fig. 2). In Ichinoseki, although the GSI average in the 13L condition was not significantly higher than that in the IG (Fig. 2 and Table S3), some of the medaka showed increased GSI (Fig. 2 and Table S2). It could be considered that the Ichinoseki population had a slightly longer critical photoperiod than the Hanamaki and Kiyosu populations. In contrast, Miyazaki and Ginoza showed growth of ovaries in response to shorter photoperiods than Ichinoseki, Hanamaki, and Kiyosu (Fig. 2 and Table S2). These two populations contained several females with large ovaries in all groups of 10L to 14L photoperiodic conditions (Fig. 2 and Table S2) and showed the ability to spawn eggs even at photoperiods of 10–11L. In the Southern Japanese group, a latitudinal cline of the critical photoperiods, that is, fish from higher latitudes required longer critical photoperiods than those from lower latitudes, was observed, except for Hanamaki. Because the Ichinoseki were collected from their natural habitat while Hanamaki was supplied from NBRP (Fig. 1 and Table S1), the difference in the critical photoperiod between these two populations may have resulted from bottleneck effects that occurred during the generations in the Hanamaki stock of NBRP.

Sawara and Egami [23] suggested population differences in response to photoperiods in medaka. Using fish collected from natural populations for their experiments, they reported that medaka collected in Hakodate showed gonadal regression after exposure to short-day conditions of 11L13D to 12L12D for a period of 27 days, whereas no significant effects of the short day on the gonad were observed in the Okinawa population. In the present study, in accord with the results of this previous study, the response to changing photoperiods was unclear in populations originating from lower latitudes, Miyazaki and Ginoza (Figs. 1a and 2). A similar phenomenon has been reported in an ecotype of the three-spined stickleback: the northern population displayed a stronger photoperiodic response than the southern population [11].

In the present study, HdrR-II1 possessed large ovaries under outdoor winter conditions (IG group in HdrR-II1 in Fig. 2 and Table S2), indicating that HdrR-II1 did not regress ovaries in response to natural environmental signals at the appropriate seasonal time. Although no egg laying was observed in HdrR-II1 outside under short-day and cold conditions, fertilized eggs were obtained during the experimental period under all photoperiod conditions from 10 to 13L under a warm temperature. Our results demonstrate that HdrR-II1 has completely lost its photoperiod responsiveness and requires an appropriate temperature for the normal regulation of reproduction. HdrR-II1 (referred to as Hd-rR) is an inbred strain [31] whose draft genome sequence has been unveiled [32] and has become a standard strain in research using medaka. Molecular phylogenetic analysis of the cytochrome *b* gene in mitochondrial DNA (mtDNA) indicated that HdrR-II1 was included in the Eastern Japan subclade distributed from the Tohoku to the Chubu area, Japan [29]. Therefore, it is reasonable to assume that the individuals from which the HdrR-II1 strain originated could respond to changing photoperiods. In the process of establishing and generating this inbred line under a long-day and warm temperature artificial environment, the photoperiodic response was not required, and gene(s) related to the photoperiodic response are thought to have mutated and lost their function. Interestingly, most laboratory mouse inbred strains also lose seasonal responses during the process of inbreeding [3, 33, 34].

### Critical temperature

Previous reports have demonstrated that ovarian maturation for reproduction in medaka requires proper water temperature [35, 36]. However, differences in critical temperatures among strains or populations have not yet been estimated. Experiment 2 demonstrated clear intraspecific differences in critical temperatures beginning with reproduction from winter conditions among local medaka populations. The ovarian development was observed at water temperatures of 20 °C in the Higashidori, 18 °C or higher in the Kiyosu, 16 °C or higher in the Hanamaki, and 14 °C or higher in the Miyazaki and the Ginoza (Figs. 3 and S1). The Higashidori and the Kiyosu showed clear critical temperatures (Figs. 3 and S1), indicating that these populations can distinguish the temperature differences of at least 2 °C. The populations from the lower latitudes, Miyazaki and Ginoza, showed lower critical temperatures under experimental conditions. In areas close to the habitat of the Ginoza population, for example, the 10-day average temperature did not decrease below 14 °C throughout the year (Nago in Fig. 1c). In addition, the day length from sunrise to sunset was 10.5 h at the winter solstice in Ginoza, Okinawa prefecture (Fig. 1b), which was a long enough photoperiod for the Ginoza to develop their ovaries in Experiment 1. Taken together with the results of Experiments 1 and 2, this suggests that the natural Ginoza females living in their original habitat may maintain reproductive capability throughout the year. These results suggest that the populations dispersed at lower latitudes extended their breeding period by gaining shorter critical photoperiods and lower critical temperatures, and adapted to the low latitudinal environment with moderate winter conditions. The tendency for lower latitudinal populations to have extended breeding seasons has also been reported in the Atlantic silverside [37] and an ecotype in the three-spined stickleback [11].

### Transplant experiment

**Fig. 5:**
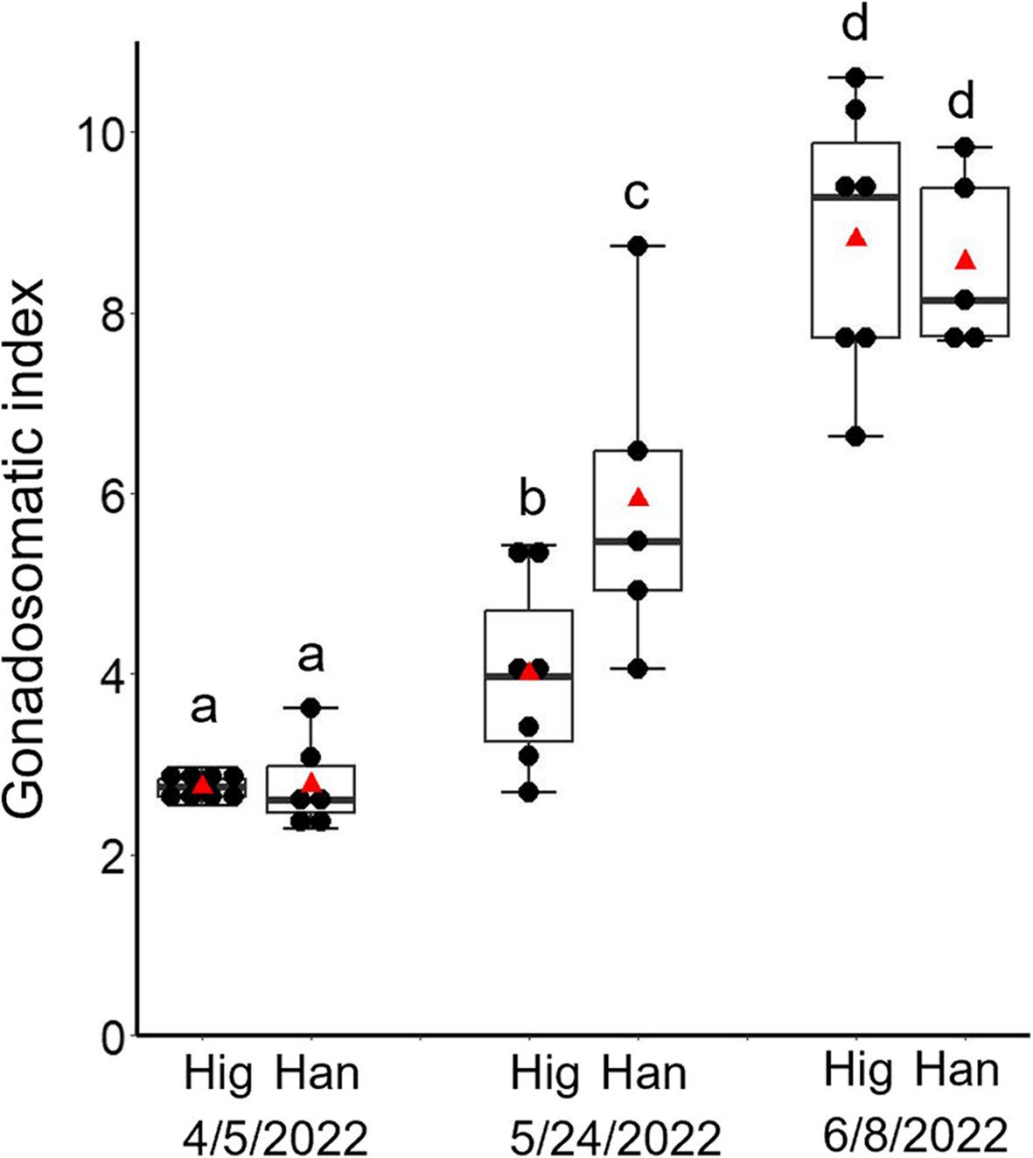
Box plot with dot plot for the GSI values of females kept at the Hakodate outdoor experimental field on April 5, May 24, and June 8. A red triangle indicates a mean value in each group. Significant differences among groups (*p* < 0.05) are indicated by a–d. The raw statistical results of two-way ANOVA and Tukey tests are shown in Tables 6 and 7

Higashidori, originating from the northernmost medaka habitat, showed the longest critical photoperiod and the highest critical temperature under artificial experimental conditions (Experiments 1 and 2). On the other hand, the Hanamaki exhibited a critical photoperiod about 1 h shorter and a critical temperature about 2 °C (4-week experiment) or 4 °C (6-week experiment) lower than Higashidori (Figs. 2, 3, and S1). We investigated the response of seasonal reproduction in these populations to a natural outdoor environment. In Experiment 3, in Hakodate, which exhibited similar annual changes in photoperiod and temperature to the Higashidori habitat (Fig. S2), the ovaries of Hanamaki developed earlier than those of Higashidori, as expected based on the results of Experiments 1 and 2. Significant differences from IG were observed on May 24 in Hanamaki and on May 31 in Higashidori (Fig. 4). Although ovaries developed approximately 1 week later in Higashidori than in Hanamaki, the time to reach a plateau in ovary size was shorter (Figs. 4 and 5). Longer critical photoperiods and higher critical temperatures result in a later initiation and earlier cessation of reproduction, leading to a shorter reproductive period. However, rapid ovarian development allows them to produce *en masse* offspring as soon as the environment becomes suitable for reproduction. In fish, there is a close relationship between body size and winter mortality; thus, sufficient growth is necessary to survive harsh winters [37]. Rapid ovarian development may compensate for the reduced growth period due to late reproductive initiation at higher latitudes. Previous reports have demonstrated that higher-latitude populations of medaka grow faster than lower-latitude populations [38, 39]. These results suggest that rapid ovarian development and growth rates are adaptations to shorter reproductive and growth periods at higher latitudes.

## Conclusion

In this study, we demonstrated differences in responses to photoperiod and temperature to initiate reproduction in female medaka among the local populations. Namely, the Northern Japanese group required a longer photoperiod and higher temperature to begin reproduction than the Southern Japanese group. Furthermore, populations originating from lower latitudes showed reduced responsiveness to photoperiod and temperature, suggesting that they have adapted to mild winter conditions by extending their breeding seasons to obtain more offspring. The extreme case is the laboratory condition without any seasonal fluctuation, and the inbred strain HdrR-II1 lost its responsiveness to photoperiod. The results of the transplant experiment in high-latitude environments supported the results obtained in artificial environments, suggesting that variations in critical photoperiod and temperature increase reproductive fitness in natural environments. Future studies on medaka populations with different critical photoperiods and temperatures will contribute to our understanding of the genetic basis of seasonal time perception.

## Supplementary Information

**Additional file 1: Supplementary Fig. S1. **Box plot with dot plot for the GSI values of females under long photoperiodic conditions (14L10D) at four different temperature conditions for 6 weeks and under the winter condition (initial group, IG) in three wild-derived populations. The raw GSI values are shown in Table S5. Black asterisks indicate significantly higher values compared to IG. *p < 0.05, ***p < 0.001. The raw statistical results of one-way ANOVA and Tukey–Kramer tests are shown in Tables S6 and S7. **Supplementary Fig. S2.** Environmental similarities between Higashidori or Mutsu and Hakodate in day length (top) and temperature (bottom).

**Additional file 2: Supplementary Table S1.** Collection sites of the medaka populations used in this study.

**Additional file 3: Supplementary Table S2. **Gonadosomatic indexes of female medaka under various photoperiods for 3 weeks in Experiment 1. 

**Additional file 4: Supplementary Table S3. ***p*-values of Tukey-Kramer tests on GSI in Experiment 1.

**Additional file 5: Supplementary Table S4. **Gonadosomatic indexes of female medaka under various temperatures for 4 weeks in Experiment 2.

**Additional file 6: Supplementary Table S5. **Gonadosomatic indexes of female medaka under various temperatures for 6 weeks in Experiment 2.

**Additional file 7: Supplementary Table S6.** F values and *p*-values of one-way ANOVA on GSI at 6 weeks in Experiment 2.

**Additional file 8: Supplementary Table S7.** *p*-values of Tukey-Kramer tests on GSI at 6 weeks in Experiment 2.

**Additional file 9: Supplementary Table S8.** Gonadosomatic indexes of female medaka in the outdoor experimental field in Hakodate in Experiment 3.

**Additional file 10: Supplementary Table S9.** Water temperature and day length in the outdoor experimental field in Hakodate in Experiment 3.

## Data Availability

All data generated or analyzed during this study are included in this published article and its supplementary information files.
