# Supplementary figures and images for: Variation in responses to photoperiods and temperatures in Japanese medaka from different latitudes
Source: Zoological Lett. 2023 Jul 22;9:16. doi: 10.1186/s40851-023-00215-8 (PMC10362753; doi:10.1186/s40851-023-00215-8)

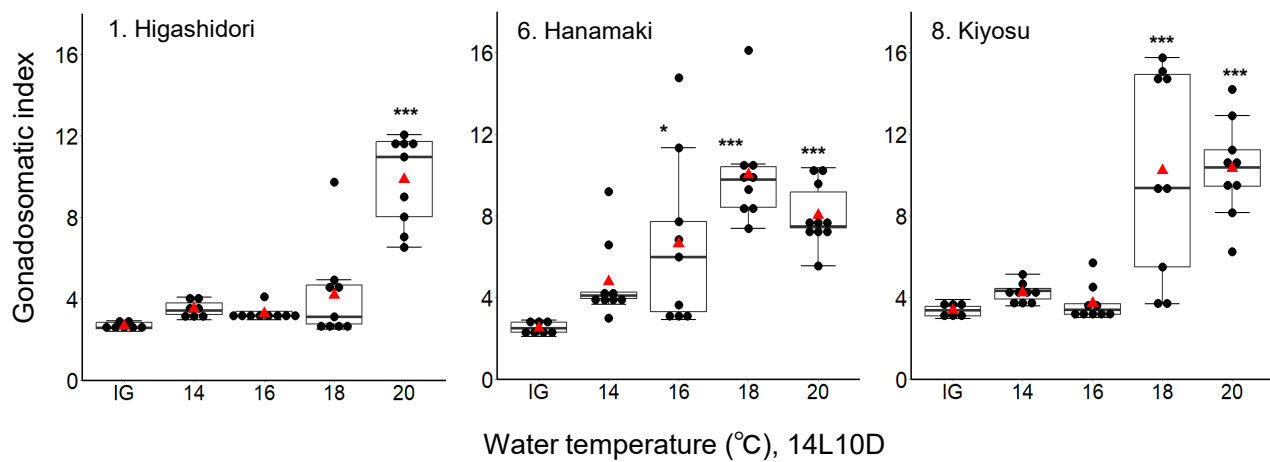

Fig. S1

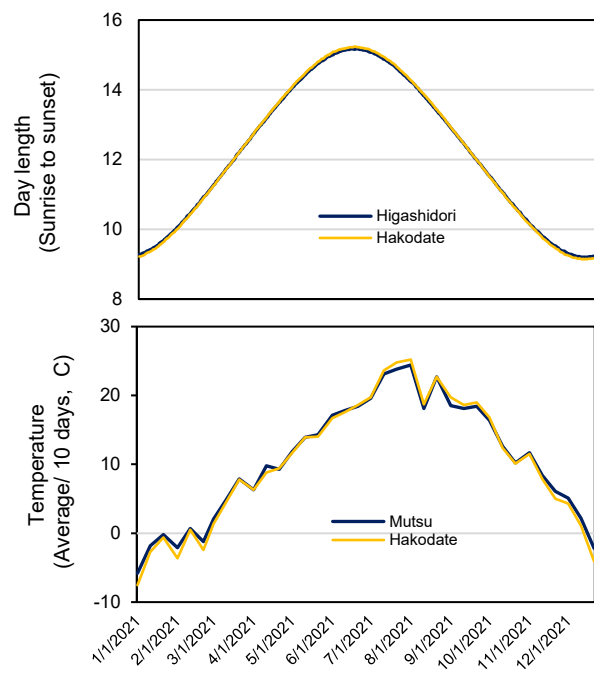

Fig. S2

Supplement: Supplementary file 1 — Additional file 1: Supplementary Fig. S1. Box plot with dot plot for the GSI values of females under long photoperiodic conditions (14L10D) at four different temperature conditions for 6 weeks and under the winter condition (initial group, IG) in three wild-derived populations. The raw GSI values are shown in Table S5. Black asterisks indicate significantly higher values compared to IG. *p < 0.05, ***p < 0.001. The raw statistical results of one-way ANOVA and Tukey–Kramer tests are shown in Tables S6 and S7. Supplementary Fig. S2. Environmental similarities between Higashidori or Mutsu and Hakodate in day length (top) and temperature (bottom). [file 40851_2023_215_MOESM1_ESM.pdf]
